# Traumatic Brain Injury Rehabilitation in Hong Kong: A Review of Practice and Research

**DOI:** 10.1155/2015/274326

**Published:** 2015-10-08

**Authors:** Junhong Yu, Helena M. K. Tam, Tatia M. C. Lee

**Affiliations:** ^1^Laboratory of Neuropsychology, The University of Hong Kong, Pokfulam, Hong Kong; ^2^Institute of Clinical Neuropsychology, The University of Hong Kong, Pokfulam, Hong Kong; ^3^The State Key Laboratory of Brain and Cognitive Sciences, The University of Hong Kong, Pokfulam, Hong Kong

## Abstract

*Background*. The rising public health concern regarding traumatic brain injury (TBI) implies a growing need for rehabilitation services for patients surviving TBI. *Methods*. To this end, this paper reviews the practices and research on TBI rehabilitation in Hong Kong so as to inform future developments in this area. This paper begins by introducing the general situation of TBI patients in Hong Kong and the need for rehabilitation. Next, the trauma system in Hong Kong is introduced. Following that is a detailed description of the rehabilitation services for TBI patients in Hong Kong, as exemplified by a rehabilitation hospital in Hong Kong. This paper will also review intervention studies on rehabilitating brain-injured populations in Hong Kong with respect to various rehabilitation goals. Lastly, the implications of culture-related issues will be discussed in relation to TBI. *Results/Conclusions*. The intervention studies conducted in Hong Kong are generally successful in achieving various rehabilitative outcomes. Additionally, certain cultural-related issues, such as the stigma associated with TBI, may impede the rehabilitative process and lead to various psychosocial problems.

## 1. Introduction

Traumatic brain injury (TBI) is a condition involving brain damage caused by external forces, such as acceleration and deceleration, impact, blast waves, or penetrating injury. Its pathophysiology is characterized by shearing of white matter tracts, intra- and extracerebral hematomas, focal contusions, and diffuse swelling [1]. This condition has affected many people around the world; every year at least 42 million people sustain a TBI [2]. These survivors of TBI are likely to suffer from many possible long-term consequences in emotional, cognitive, and daily functioning [3–5].

Similar to findings from studies conducted in Western populations [6], survivors of TBI in Hong Kong also report similar consequences following their brain injury such as having a low quality of life. Specifically, they are unsatisfied with their material well-being, their place in the community, and their productivity at work [7]. This dissatisfaction may very likely be due to their reduced ability to work to support themselves and thereby contribute to the community. In fact, the postinjury employment rate of brain-injured individuals in Hong Kong ranges from 10% to 47% [7, 8], which is relatively low compared to their Western counterparts [9]. Another possible reason for their low quality of life may revolve around interpersonal issues commonly affecting survivors of TBI. Those who had been living with brain injuries for an extended period of time (≥5 years) reported low satisfaction in the domain of intimacy [7], suggesting that they have experienced some strained relationships with others. The aging population in Hong Kong [10] further complicates the local TBI context in two ways. First, the elderly population is highly prone to falls [11] and falls are the most common cause of trauma-related injuries within the Hong Kong context [12]. Hence, as the population grows, we would expect the incidence of TBI to increase correspondingly. Second, relative to younger brain-injured patients, elderly patients require longer hospitalizations and have poorer functional outcomes [13, 14].

According to Yeung et al. [15], in Hong Kong approximately a fifth of all emergency cases received by hospitals were trauma related, and more than half of these cases were diagnosed with TBI [12]. The increasing incidence of TBI is paired with a growing need and importance for rehabilitative services catered to those afflicted with TBI. TBI patients, even those who suffer from mild but repetitive TBI, may suffer long-term consequences, such as progressive brain atrophy and increased vulnerability to neurodegenerative disorders [16]. Within the cognitive domain, those with moderate to severe TBI are likely to be impaired in attention, memory, executive functions, and insight relating to their deficits [4, 17]; these impairments will in turn have an adverse impact on functional outcomes [18]. Those with mild TBI are not spared from cognitive impairments either; they may still report clusters of attentional impairments and deficits in verbal fluency [19] despite sustaining the injury years ago [20]. They also experience difficulties in the psychosocial domain—often as a result of a combination of injury-related, psychological, and social factors [21, 22], which predisposes them to a range of psychosocial and emotional problems [5]. Taken together, these consequences also hinder their reintegration into the community [23]. Fortunately, some of these consequences are at least amenable to rehabilitation. In a review of randomized controlled trials (RCTs) targeting various rehabilitation goals in patients with moderate to severe TBI, 36 out of 45 RCTs reported significant positive gains in the cognitive, functional, and/or psychosocial domains [24]. The need for rehabilitative efforts to better the lives of TBI patients cannot be understated; rehabilitation for TBI is a key area to direct research and intervention efforts so as to better patients' outcomes in Hong Kong. While there has been extensive work in the literature on the rehabilitation of TBI patients, most of this research has been conducted with Western populations and in managed care settings; research in the Asian contexts is lacking. Moreover, Asian cultural factors in TBI rehabilitation have often been overlooked in the literature. Given that health care infrastructure and culture are major factors in shaping rehabilitative efforts [25, 26], there might be issues with translating these findings into practice in Hong Kong—a society with a predominantly Chinese population where healthcare services are typically financed by government subsidies and out-of-pocket payments (by the patients and/or their families), instead of relying on social or private insurance. Hence, whenever possible, it may be more useful to look at the practice and research in the local context, so as to inform local TBI rehabilitation efforts. To this end, the current report aims to review the practice and research of rehabilitating TBI patients in Hong Kong as well as explore the implications of TBI in the local cultural context. The present review is focused only on the rehabilitation of the adult TBI population (i.e., aged 18 years and above) in Hong Kong.

## 2. Description of TBI Practices/Health Care Services/Rehabilitation in Hong Kong

### 2.1. Trauma System

**Figure 1:**
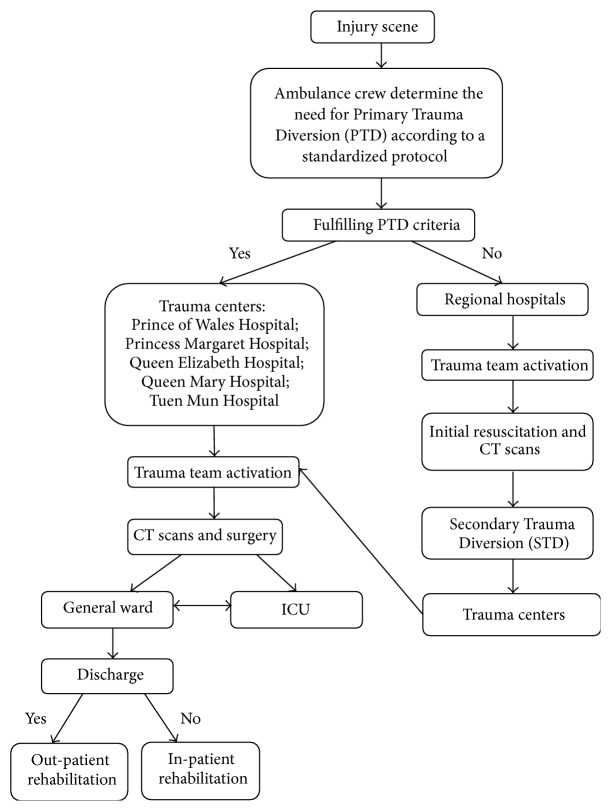
Flow diagram illustrating trauma patient management.

Patients who sustain trauma-related injuries are served by the trauma system as managed by the Hospital Authority (HA). The current trauma system, comprising five regional trauma centers—namely, Prince of Wales Hospital, Princess Margaret Hospital, Queen Elizabeth Hospital, Queen Mary Hospital, and Tuen Mun Hospital—was set up gradually in 2003 to provide trauma care services to various hospital clusters. For instance, Queen Elizabeth Hospital, which is based in central Kowloon, serves one such cluster by extending its trauma services to three other hospitals within its close proximity [27]. These five trauma centers had met the criteria for a Level I trauma center and were well equipped to handle trauma cases [28]; a Level I trauma center has to be able to admit at least 1,200 trauma patients or receive 240 admissions of severe injuries in a year [28]. In the past, trauma casualties were brought to the nearest public hospital regardless of the severity of their injuries. With the pilot implementation of the Primary Trauma Diversion Policy in 2003, trauma casualties are transported from the scene to these trauma centers directly if necessary, as determined by the ambulance crew according to a standardized protocol [29]. Alternatively, the crew may decide to transport the casualty to a regional hospital for initial resuscitation before transferring to a trauma center, that is, a Secondary Trauma Diversion [30]. Prehospital trauma care is provided by the ambulance services; the paramedics were trained and equipped to provide such care on the way to the hospital [31]. Subsequently, a trauma team consisting of an emergency physician, trauma surgeon, and other specialists [32] is called upon to attend to the case received at the trauma center [33]. These trauma centers provide a variety of services for the acute care and rehabilitation of TBI patients. On top of that, there are a few convalescent hospitals that specifically cater to their rehabilitative needs, such as Tung Wah Eastern Hospital, MacLehose Medical Rehabilitation Centre, Rehabid Centre, Rotary Rehabid Centre for Children, and The Duchess of Kent Children's Hospital. These rehabilitation units provide a range of services for neurological patients in general. However, due to limited resources, some patients with TBI may also receive rehabilitation services from other hospitals.  Figure 1 depicts the flow of trauma case management.

### 2.2. Rehabilitation

For the purpose of describing the rehabilitation of TBI patients in Hong Kong, we shall examine a rehabilitation hospital in Hong Kong: the MacLehose Medical Rehabilitation Centre (MMRC) [34]. This example was selected due to its significance of being the first and only rehabilitation hospital opened on Hong Kong Island. In this hospital, rehabilitation regimes are tailored according to each patient's assessment findings and are delivered by a multidisciplinary team. For TBI cases, this team would usually include clinical psychologists, occupational therapists, physiotherapists, and speech therapists, in addition to other medical professionals. Cognitive rehabilitation is conducted by the clinical psychologists. Based on the concept of neuroplasticity, well-structured cognitive training programs, targeting attention, memory, visual-spatial, and executive functions, are delivered to TBI patients. The occupational therapists assist the patients to work on their self-care skills and household management and modification, as well as community integration. The physiotherapy team implements a rehabilitation program to maximize the patients' existing physical abilities and to regain functional independence. For this purpose, they are equipped with a neurological room, a standard sized gymnasium, and a hydrotherapy room. The speech therapists attend to patients who have issues with swallowing and/or verbal communication. These different specialists work closely with each other to contribute to the rehabilitation process and to carry out interventions tailored to each patient. They also meet up in weekly case rounds to discuss the progress of each patient. After a patient has completed his/her assigned rehabilitation regime, predischarge arrangements are made by the same team.

Patients usually receive short-term rehabilitation services in hospital settings. They may however, with an appropriate referral, turn to various nongovernmental organizations (NGOs) in Hong Kong for long-term care and rehabilitation. These NGO rehabilitation centers provide various outpatient services for discharged neurological patients and also facilitate the patient's reintegration into the community.

## 3. Review of TBI Rehabilitation Research in Hong Kong

At the time of this writing, there have not been any published trials on the rehabilitation of, specifically, TBI patients in Hong Kong. Given the paucity of TBI research in Hong Kong, it might be worthwhile to broaden the scope to other brain-injured populations, such as that of stroke. Stroke and TBI are highly similar in terms of pathophysiology. Hence, rehabilitation strategies beneficial to patients with stroke are likely to be beneficial to those with TBI as well [35]. For the present review, literature search was conducted using PubMed and Scopus with the following search terms in the title or abstract of the article: traumatic brain injury, TBI, close head injury, stroke, intervention, RCT, trial, training, remediation, rehabilitation, and Hong Kong. The exclusion criteria were (1) nonintervention studies, (2) studies that were not conducted in the postacute phase of the brain injury, (3) studies that did not include brain-injured participants, (4) studies that included participants below the age of 18 years, (5) studies that were conducted outside Hong Kong, and (6) studies that did not focus on functional related outcomes or had focused on narrow areas of physical functioning (i.e., hemiparetic upper extremity). A total of 7 studies were retrieved with the above criteria.

These studies have achieved various significant rehabilitative outcomes among brain-injured patients in Hong Kong via different modalities and objectives. For instance, improving the daily functioning of these patients is one such objective; these patients are likely to be impaired in many aspects of their daily functioning [36, 37]. To remediate such impairments, two interventions utilized relearning protocols to enhance competency on tasks with which patients had difficulty with following stroke. In the first, the intervention utilized mental imagery to achieve patients' goals [38]. Patients (*n* = 49) were randomly assigned to the mental imagery group or functional retraining group for three weeks (5 h/wk). In the mental imagery group, patients were taught to use mental imagery to analyze and identify their difficulties and practice certain tasks. The functional retraining group practiced similar tasks; however, unlike the mental imagery group, the entire process was more instructional and didactic and did not involve mental imagery. At the end of the intervention, the mental imagery group had significantly higher levels of task performance on both trained and untrained tasks.

The other relearning intervention focused on the relearning of motor skills [39]. A total of 66 patients were randomly assigned to a motor-relearning program or conventional therapy program for six weeks (6 h/wk). The motor-relearning program involved a step-by-step process of first identifying one's motor task-related deficit, remediating this deficit by practicing on selected tasks related to the deficit, and transferring the skills acquired to functional tasks. The conventional therapy program was similar, except that the task selection was based on the patients' physical status rather than their deficits and patients were not trained to identify their deficits. At the end of six weeks, group × time interaction effects suggested that the motor-relearning program had produced better outcomes relative to the conventional therapy program on measures of functional balance, self-care ability, daily living tasks, and community integration.

Stroke and TBI patients may both experience problems related to mobility [3, 40]; hence, it is important for community-based interventions to reach out to these people, who may have difficulties accessing rehabilitative facilities far away from home. There were three studies that specifically tackled such issues. The first study [41] made use of home visits within a nurse-led transitional care RCT. Stroke patients (*N* = 108) were randomly distributed between the intervention and control groups. The intervention group was administered a holistic care package that was delivered via weekly home visits that involved motivational interviewing and follow-up telephone calls for four weeks. Subsequently, the intervention group reported better physical functioning relative to controls immediately after the intervention and at a follow-up assessment four weeks later.

The second study [42] had video conferencing apparatus sited at various community venues in a district to reach out to their participants. Stroke patients (*N* = 21) took part in an eight-week intervention (1.5 h/wk) that consisted of stroke education and physical exercise components, both of which were delivered via video conferencing to all participants. At the end of eight weeks, there were significant increases, relative to baseline, in measures of physical balance ability, self-esteem, quality of life, and knowledge of stroke. It should be noted that these results should be interpreted conservatively since there were no comparisons to a control group.

The third study [43] examined the option of having a short-term residential care program to eliminate the need for patients to travel. In this study, 188 stroke patients were assigned either to the residential care program or to a usual care program (as similarly described in the previous section) in a public hospital (both 12 h/wk). The residential care program had similar rehabilitation provisions as the usual care program, though their rehabilitation regime was spread out across more days of the week. Additionally, in the former, participants stayed in a home-like environment instead of hospitals and received round-the-clock residential and nursing care. At the end of four months, both groups had similar levels of improvements in general cognition, daily functioning, caregiver burden, depression, and self-esteem.

Brain injuries can also result in a variety of cognitive deficits as mentioned earlier. Memory impairments are one of these deficits that was of key interest in Tam and Man's [44] intervention study. In their study, 34 brain-injured patients with impairments in semantic memory were assigned to four different computerized training groups for two weeks (2.5 h/wk) and a no-treatment control group. Those in the training groups were trained to remember similar contents across groups, such as faces and names, things to do, something that was said, and where to place an item. However, the training strategy differed across groups: The first group was allowed to work at their own pace; the second group was given clear, consistent, and nonjudgmental feedback at every instance; the third group made use of actual stimuli that the participants had seen or known before; and the last group utilized attractive and bright stimuli. Even though all four training groups showed significant performance improvements across time on the computerized tasks within their training programs, none of the training groups reported significant improvements across time on a standardized memory assessment (i.e., Rivermead Behavioural Memory Test).

Attention is another cognitive domain where intervention work could be targeted. In one such experiment [45], 10 patients with closed head injury (CHI) were compared with 10 healthy controls on jigsaw puzzle tasks. All participants completed two different jigsaw puzzles while being recorded on video. In one of the puzzle tasks, they were instructed to verbalize their actions, and in the other no such instructions were given. Also, in the course of completing a puzzle, distractors (such as someone dropping a book or playing on the computer) were introduced. The authors divided the recorded videos into fifteen-second clips in which they coded for the presence of a distractor and the presence of off-task behaviors (e.g., head and eyes oriented away from the puzzle). The numbers of correct and incorrect placements of the puzzle pieces were also recorded. The results indicated that verbalization did have a significant effect on reducing the number of off-task behaviors in the presence of distractors in both CHI patients and controls. It was also reported that there were significantly more correct placements in the verbalization condition than the nonverbalization condition in both groups. In both outcome measures, the differences attributed to verbalization were larger for CHI patients than for controls. The authors, while noting possible ceiling effects on the controls, suggested that CHI patients, relative to controls, can benefit more from such verbalization techniques.

The results of these intervention studies in Hong Kong are generally consistent with previous research in showing that functional outcomes among the brain-injured, such as those within the physical, psychosocial, and cognitive domains, can be improved via various rehabilitative approaches [24]. However it should also be noted that most of these studies do not meet Cicerone et al.'s [46] criteria of a well-designed “Class I” study for their findings to be translated into practice standards and guidelines.

## 4. Cultural Implications of TBI in the Local Hong Kong Context

The disabilities or dysfunctions associated with TBI may not be the only concerns of TBI patients. TBI patients may experience certain psychosocial problems, which have less to do with the severity of the injury but perhaps more so to do with societal perceptions [47]. These societal perceptions or stigma may also have major implications for TBI patients' rehabilitation. The discussion of stigma has a special relevance here in Hong Kong, given the cultural context and the nature of the public health system.

One cannot easily identify a TBI patient just from his/her appearance because TBIs are usually not associated with any physically obvious abnormalities unlike patients with physical disabilities. Hence, individuals with TBI can choose whether to conceal their disabilities or disclose them to others. In either scenario, there will be undesirable consequences. If they disclose their condition to others, they might risk being stigmatized by the society as being mentally ill [47]. This is due to a number of reasons: (1) similar to those afflicted with mental illness, TBI patients do sometimes exhibit deviant behaviors and mental disabilities/impairments as well [48], and as a result, the layperson may not be able to distinguish between neurological conditions and mental illnesses [49]; (2) TBI patients are often diagnosed with mental illness following TBI [50]; and (3) the label of “brain-injured,” like “mentally ill,” is equally reductionistic and carries similar negative connotations for the individual [47]. Furthermore, this stigma can be particularly unpleasant in Hong Kong because of the traditional Chinese belief that mental illness reflects the inferiority of one's family, the failure of one's parents, and the misdoings of one's ancestors [51]. In essence, having a mentally ill relative is something to be ashamed of in the Chinese context. This stigma or shame adds further stress to the family, who are already burdened by the need to provide and care for their brain-injured relatives. Within Chinese contexts, families assumed the primary role in the management, coordination, and provision of care for TBI patients. However, these families were generally not adequately equipped in terms of resources and information to deal with the challenges of caring for their brain-injured relatives. As a result of undertaking these responsibilities, most of these families are already overwhelmed with shock, negative emotions, and uncertainty and consequently compromised their own physical and psychological well-being in the process [52, 53].

As a result, it is likely that TBI patients, like those diagnosed with mental illness, would choose to conceal their conditions from others to avoid the stigma or even hide their condition from their family members to avoid implicating them [54]. In fact, this tendency to cover up has been documented among TBI patients from a similar cultural background in a qualitative study. In this study, Vietnamese respondents reported attempting to cover up or not draw attention to their TBI by avoiding friends and telling lies because they were worried it might bring shame to their families [26]. This culture of concealing one's condition makes it difficult for people with TBI to seek social support, access rehabilitative services, and receive social welfare benefits. In Hong Kong, TBI is considered as a form of disability and TBI patients—like those who are physically disabled—may apply to receive social welfare assistance to cope with their disability. However, in order to obtain these benefits, one has to be certified “severely disabled” by the authorities [55], and this label may deter TBI patients from applying for these benefits especially if they would prefer to not draw attention to their conditions or disabilities. Furthermore, by concealing their disabilities, TBI patients receive less sympathy and empathetic understanding for their inappropriate actions and deficits, and as a result, unrealistic expectations are often placed upon them at the workplace based on the failure to compensate for their disabilities [56]. Even among family members who are well aware of the patient's condition, their lack of understanding of the residual impairments of TBI will similarly result in such unrealistic expectations. For instance, family members and caregivers would assume a hospital discharge implies that the patient will be able to revert back to his normal life, such as by returning to work, especially since the patient does not present with any obvious physical abnormalities that might indicate functional impairments. These unrealistic expectations set upon individuals with TBI will further complicate their interpersonal relationships and occupational functioning. The failure to satisfy such expectations will adversely affect the patient's self-concept as well. This is especially so in the Chinese culture whereby having a job symbolizes good health [8], and work itself is a major aspect of one's self-concept [57].

Taken together, the stigma encountered by TBI patients can hinder their rehabilitation in more than one way. This is a major concern that has yet to be adequately addressed in the current practices and research on rehabilitating TBI patients in Hong Kong. Certainly, more effort should be devoted to tackling such stigma. One effective way in which this can be accomplished is to promote more interpersonal contact with and exposure to this group of people. This method was found to be more effective than education alone in reducing various aspects of the stigma and can be implemented widely via school-based programs [58].

## 5. Future Directions

The current rehabilitation practices for TBI patients in Hong Kong have generally satisfied the guidelines for the rehabilitation following acquired brain injury as laid out by the Royal College of Physicians and British Society of Rehabilitation Medicine [59]. TBI patients in Hong Kong were also generally satisfied with the rehabilitative services provided [7]. In particular, they were most satisfied with the services catering to their physical well-being and functioning (such as medical services, occupational therapy, and physiotherapy); these services were also rated as highly important. On the other hand, while vocational counseling was ranked as one of the most important needs, it was also one of the least satisfactory services provided to them [7]. This, taken together with the economic, psychological, and cultural implications of returning to work discussed previously, suggests a critical need to improve rehabilitative efforts geared towards the reemployment of TBI patients. Certainly, more research and resources can be directed towards enabling TBI patients to recover their preinjury and maximize their postinjury occupational ability.

Additionally, given that the patients' main caregivers are their families, it is crucial to involve them in the rehabilitation process prior to the patient's hospital discharge. However, such family involvements remain somewhat low, and perhaps this explains their unpreparedness and lack of the know-how in caring for their brain-injured relatives [52, 53]. The fact that TBI patients' disabilities may not be reflected in any atypical physical features (as discussed previously) makes this worse; these families may sometimes have unrealistic expectations of their brain-injured relatives upon discharge simply because they “look fine.” As a result, tensions between the patient and his/her family members may arise, making it more difficult to care for the patient. Hence, to minimize such problems, prior to the patient's discharge, it would be helpful to inform and educate these families on the patient's impairments and the appropriate way to handle these impairments and care for the patient.

The few reported researches on interventions for brain-injured populations conducted in Hong Kong have generally been effective in achieving certain specific goals such as the remediation of skills compromised by the injury and community integration. Despite the lack of local research on specifically rehabilitating TBI patients, these researches on other brain-injured populations do suggest that there are adequate resources, both in the community and in the health care infrastructure, to carry out similar rehabilitative efforts on TBI patients.

Certainly, future research on TBI rehabilitation in Hong Kong will be useful in informing and guiding the current practices and ultimately to better the lives of TBI patients. Intervention efforts utilizing cognitive-based rehabilitative approaches on brain-injured populations in Hong Kong have been scarce. In light of the possible impairments in various cognitive domains [18] and the fact that these impairments are a major concern among discharged brain-injured patients in Hong Kong [53], there is huge potential for intervention work in this area. Additionally, with advances in neuroimaging, it will be useful for future interventions to track neuroplastic changes in the brain as a function of the intervention progress, so as to gain a better understanding of the brain mediators/mechanisms of such improvements in cognition, especially within the context of a neurological condition like TBI.

## 6. Conclusions

The present paper has reviewed the practice and research of TBI rehabilitation in Hong Kong. TBI rehabilitation in Hong Kong is carried out by a multidisciplinary team of health care professionals primarily within hospitals, as consistent with international guidelines. Despite limited resources, these rehabilitation services have generally satisfied most of the TBI patients' needs. However, there is room for improvement in rehabilitative efforts aimed at enabling them to return to work. While stigma is a major concern for these patients and their rehabilitation, it is hardly addressed in the current practices. Finally, research on TBI rehabilitation in Hong Kong is lacking; there is a need for more local research in this area to inform the current practice of TBI rehabilitation and more importantly to benefit TBI patients in Hong Kong.
